# Serum Levels of Hormones Regulating Appetite in Patients with Fetal Alcohol Spectrum Disorders

**DOI:** 10.3390/nu15194215

**Published:** 2023-09-29

**Authors:** Rafał Podgórski, Sabina Galiniak, Artur Mazur, Dominika Podgórska, Agnieszka Domin

**Affiliations:** 1 Department of Biochemistry, Institute of Medical Sciences, Medical College of Rzeszow University, 35-310 Rzeszow, Poland; sgaliniak@ur.edu.pl; 2 Department of Pediatric, Institute of Medical Sciences, Medical College of Rzeszow University, 35-310 Rzeszow, Poland; drmazur@poczta.onet.pl (A.M.); adomin@ur.edu.pl (A.D.); 3 Department of Rheumatology, Institute of Medical Sciences, Medical College of Rzeszow University, 35-310 Rzeszow, Poland; dpodgorska@ur.edu.pl

**Keywords:** neuropeptide Y, Agouti signaling protein, alpha-melanocyte-stimulating hormone, kisspeptin, FASD

## Abstract

Prenatal alcohol exposure is the cause of impaired growth and a wide range of developmental and behavioral disorders in the child. Improper eating patterns are commonly associated with fetal alcohol spectrum disorders (FASD) and may contribute to poor nutrition and growth restriction. To date, there have been only a few studies investigating the hormonal regulation of appetite in patients with FASD. We analyzed the levels of neuropeptide Y (NPY), Agouti signaling protein (ASP), alpha-melanocyte-stimulating hormone (α-MSH), and kisspeptin (KISS1) in 57 patients with FASD and 23 healthy controls. A comparison of the hormone levels studied was also performed in subgroups of fetal alcohol syndrome (FAS) and neurobehavioral disorder associated with prenatal alcohol exposure (ND PAE), as well as in males and females. We have found no differences in hormone levels tested between affected individuals and the controls and between FASD subgroups. In addition, sex had no effect on hormone levels. However, we identified some associations between hormone concentrations and parameters describing the clinical status of patients with FASD. Most of them concerned ASP, which has shown a positive correlation with age and hormones involved in appetite and metabolism, such as proopiomelanocortin (POMC) and adrenocorticotropic hormone (ACTH). We have also found a negative correlation of α-MSH with age, BMI percentile, and glycated hemoglobin (HbA1c). Furthermore, we found a weak negative correlation of NPY with HbA1c. Although FASD has been associated with impaired child growth and development, including nutrition and puberty onset, we did not identify differences in the levels of the hormones studied, which may suggest that prenatal alcohol exposure does not affect the levels of these metabolites.

## 1. Introduction

The formation and development of the brain occurs during the fetal and infancy period. The teratogenic effect of alcohol on the fetus is now well established [1,2]. Fetal exposure to alcohol increases the risk of stillbirth, impaired growth, and a wide range of developmental, physical, and cognitive disorders in the growing child [3]. Specific minor facial structures and somatic growth retardation are characteristic features of prenatal alcohol exposure. However, the most significant is its effect on brain development, which causes substantial problems in neurobehavioral development. The syndrome resulting from prenatal alcohol exposure is called fetal alcohol spectrum disorder (FASD) and includes fetal alcohol syndrome (FAS), partial fetal alcohol syndrome, alcohol-related neurodevelopmental disorder, and alcohol-related birth defects [4].

Alcohol was recognized as harmful to the developing child in utero more than 50 years ago, but it still remains a real social and health problem despite the growing evidence and knowledge on this subject. [5]. Recent data have shown that the global prevalence of FASD among children and adolescents in the general population was estimated at 7.7‰ [4]. Moreover, the scale of the problem has not decreased but is growing as well. In some regions, such as New Zealand, Australia, and the United Kingdom, the highest prevalence was 19.8‰, and these numbers are even higher among low socioeconomic populations [6,7]. Furthermore, children with a history of prenatal alcohol exposure and less severe symptoms of FASD often go undiagnosed or are misdiagnosed, so these actual prevalence rates are likely underestimated [8]. Making a diagnosis, especially the milder forms, in children and adolescents is difficult due to the lack of unified and standardized criteria, the frequent lack of awareness in the child’s family of the possible consequences of alcohol abuse for the fetus, and the fact that alcohol consumption is often subject to a strong social taboo even for physicians [9]. Additionally, approximately 50% of pregnancies are unplanned, and a woman may not know she is pregnant until six weeks or more and still consume alcoholic beverages. The risk of FASD throughout pregnancy is variable and depends on the duration and frequency of alcoholic intoxication [3]. Exposure of a fetus to alcohol at different stages of pregnancy increases this risk respectively: 12 times in the first trimester, 61 times in the first and second trimesters, and 65 times in the entire pregnancy [10]. There is no known safe and allowable amount of alcohol during pregnancy, so complete abstinence is recommended to avoid the risk of developing FASD [11].

Children prenatally exposed to alcohol show great variability in dysmorphic and neurological features. Neurological deficits involve cognitive and executive functions, and impairments in memory, hearing, vision, motor skills, behavior, and social adaptation [12]. FASD is the most common possible preventable cause of neurodevelopmental disorders worldwide [13]. Fetal and long-term growth restriction is well established in FASD and is directly related to the quality and quantity of alcohol exposure. Heavy exposure results in intrauterine and postnatal growth restriction, which affects all parts of the body, including the brain, and is correlated with the severity of cognitive impairment [9]. Furthermore, improper eating behaviors are common in children with FASD and may contribute to poor nutrition and growth restriction [14]. It is well known that in infancy and early childhood, FASD is strongly linked to lower than normal head circumference, height, and weight [15]. These discrepancies decrease in later childhood, and even faster weight gain and overweight are observed in people with FASD, starting in puberty [16,17,18]. Nutritional control is a very complex process that is regulated at multiple levels, such as the central nervous system (CNS) and peripherally, by hormonal signals released by the gut and adipose tissue describing the energy state of the body [19]. Some of the hormones involved in the regulation of nutrition in CNS are neuropeptide Y (NPY), Agouti-signaling protein (ASP), alpha-melanocyte-stimulating hormone (α-MSH), and kisspeptin (KISS1). NPY and ASP are orexigenic hormones, whereas α-MSH and KISS1 inhibit appetite [20].

The objective of the study was to assess the level of four hormones (NPY, KISS1, ASP, α-MSH) involved in appetite regulation in children with FASD and to compare these values with a control group. To our knowledge, this is the first study to describe the levels of these hormones in patients with FASD. In addition, we investigated their relationship with other hormones that regulate appetite and metabolism. Proopiomelanocortin (POMC) is a precursor hormone that gives rise to biologically active peptides that are expressed in the brain. These include adrenocorticotropic hormone (ACTH), α-MSH, β-lipotropin, and endorphins. ACTH is the factor that controls the release of cortisol, the main glucocorticoid that, among other things, regulates energy and metabolic processes.

## 2. Materials and Methods

Study group. A study was performed on 57 FASD patients and 23 healthy controls. The group of affected individuals was divided into two subgroups: FAS and ND-PAE (neurobehavioral disorder associated with prenatal alcohol exposure). The diagnosis was made according to the most recent Polish guidelines [21]. In terms of accepted guidelines applied internationally, the ND-PAE domain includes partial fetal alcohol syndrome and alcohol-related neurodevelopmental disorder [12,22,23,24]. The study protocol was authorized by the Bioethics Committee of the University of Rzeszow (16/02/2019). Informed consent was obtained from all participants or a parent and/or legal representative. All affected participants were treated at the outpatient clinic and/or Department of Pediatrics, Endocrinology and Pediatric Diabetology at the Clinical Provincial Hospital in Rzeszow. The study was carried out from March 2019 to January 2022. Forty-four patients with FASD were excluded from the study group due to a lack of informed consent and/or use of medical records for study purposes. The difficulties were mainly due to the social situation of the patients and the epidemic situation during the project. In addition, five children diagnosed as at risk for FASD but not confirmed to have FASD were excluded from further analysis.

Hormone level determination: Neuropeptide Y, Agouti signaling protein, kisspeptin, and alpha-melanocyte-stimulating hormone were determined in serum samples. Blood was obtained from patients in the morning, on empty stomachs and serum collected after centrifuge (1500× *g*, 4 °C, 10 min), partitioned and preserved at −80 °C. Serum hormone levels were measured in duplicate with appropriate dilution using commercially available enzyme-linked immunosorbent assays (Wuhan Fine Biotech Co., Ltd., Wuhan, China) in accordance with the manufacturer’s protocol. The limit of detection for NPY (catalog number- EH4041) was 9.375 pg/m, ASP (catalog number- EH13961) was 0.094 ng/mL, α-MSH (catalog number- EH0792) was 7.5 pg/mL, KISS1 (catalog number- EH2126) was 0.094 ng/mL, and the within-assay and between-assay coefficient of variations were lower than 8% and lower than 10%, respectively. Adrenocorticotropic hormone (ACTH) and cortisol were determined by the chemiluminescent microparticle immunoassay method on an Alinity analyzer (Abbot, Abbott Park, IL, USA). Blood morphology was determined using a hematology analyzer (Siemens Healthineers, Erlangen, Germany).

**Statistical analysis.** Statistical analysis was performed using the STATISTICA software package (version 13.3, StatSoft Inc. 2017, Tulsa, OK, USA). Data were presented as mean and SD or median, as well as range). The distribution of variables was assessed by the Shapiro−Wilk W test, and due to most of them not following normal distribution nonparametric methods, the Mann−Whitney U test was applied for comparison between two independent groups. A *p*-value less than 0.05 was considered statistically significant. The correlation between variables was evaluated using the Spearman rank correlation test, assuming linear dependence with α = 0.05.

## 3. Results

A total of 57 affected individuals with FASD participated in the study, including 29 females and 28 males. Furthermore, 23 healthy children were enrolled in the study (16 males and seven females (30.5%)). The most important anthropometric parameters and basic laboratory of patients with FASD and healthy controls are presented in Table 1.

Basic demographic and anthropometric measurements have not shown age differences between patients with FASD and healthy controls (*p* = 0.526). Age also did not differ between the FAS and ND PAE subgroups (*p* = 0.843) (Table 2). The BMI percentile values were significantly lower in the FASD group compared to the control group (*p* = 0.037) as well as in the FAS subgroup compared to ND PAE (*p* = 0.020).

In the FAS subgroup, children with low height (<3 percentile) accounted for 42.31%, while in the ND-PAE subgroup, children with low height comprised 18.18%. There were no significant differences between the compared groups in biochemical parameters such as lipid profile, glucose, insulin levels, or HOMA-IR. Statistically significant differences were observed only with respect to HbA1c (%), which was lower in the FAS subgroup (*p* = 0.039).

**Figure 1:**
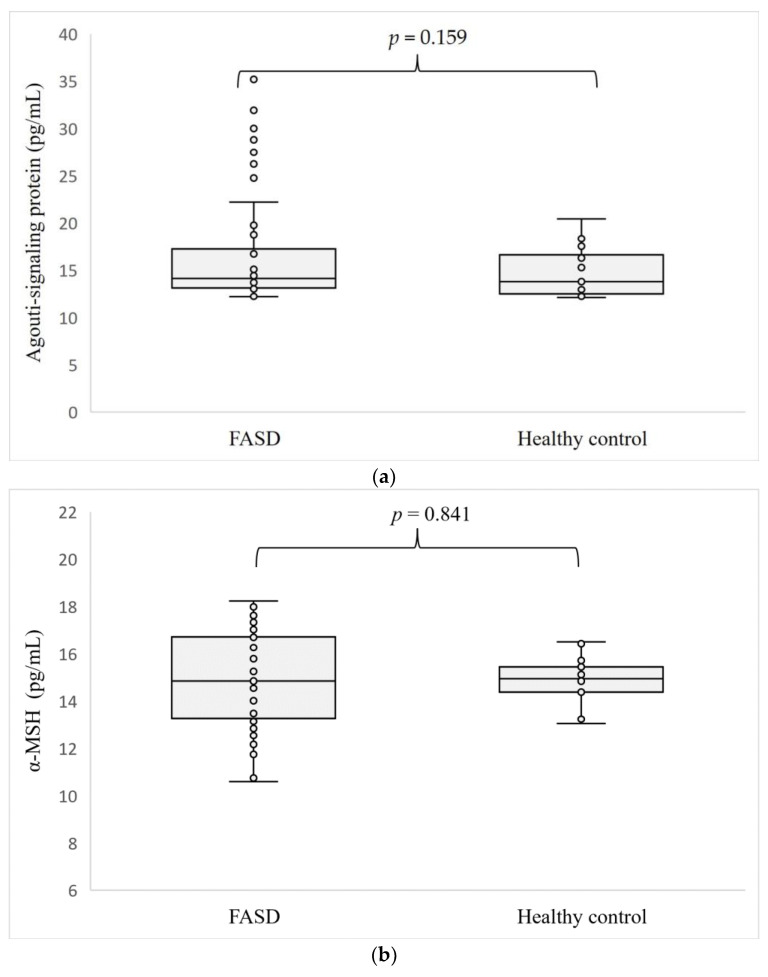

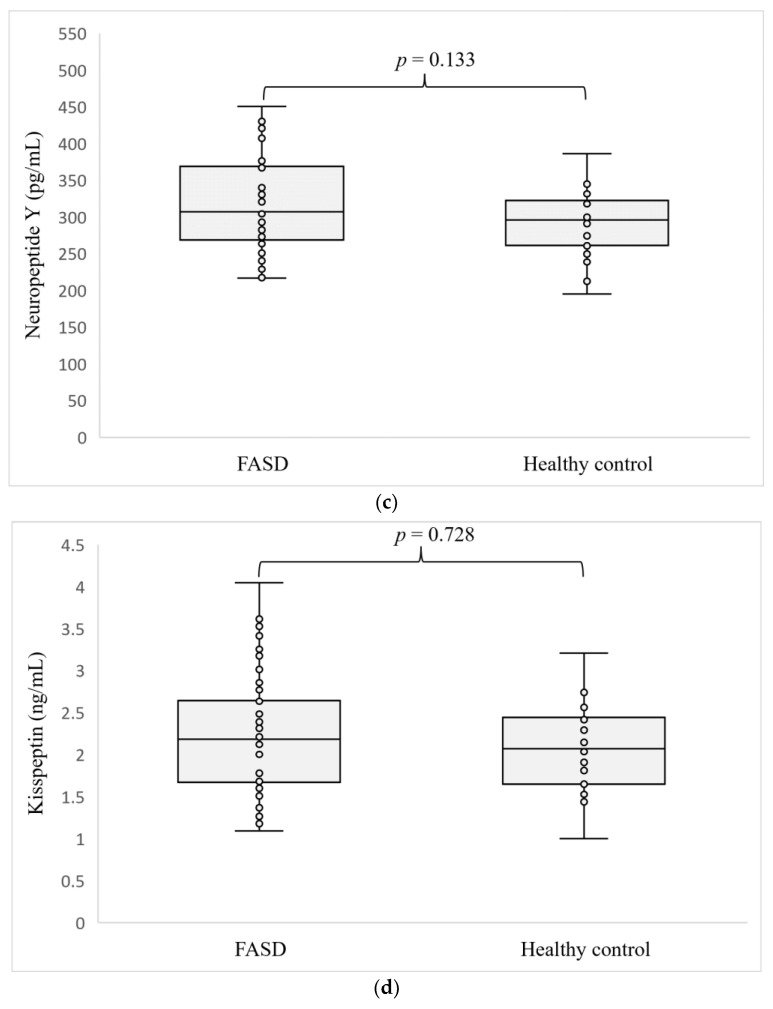
Level of ASP (**a**), α-MSH (**b**), NPY (**c**), and KISS1 (**d**) in patients with FASD compared to healthy participants.

The levels of the hormones determined are shown in Figure 1. Concentrations of all four hormones tested: ASP (*p* = 0.159), α-MSH (*p* = 0.841), NPY (*p* = 0.133), and KISS1 (*p* = 0.728) were at similar levels in the serum of patients with FASD compared to healthy individuals.

Furthermore, a comparison of ASP, NPY, α-MSH, and KISS1 levels between FASD subgroups showed no significant differences. However, we can see a trend in the comparison between FAS and ND-PAE for α-MSH (*p* = 0.071), the levels of which seem to be higher in FAS (Table 3).

Comparisons of hormone levels in females and males with FASD are presented in Table 4. We did not observe any differences in concentrations of ASP (*p* = 0.856), α-MSH (*p* = 0.596), NPY (*p* = 0.173), and KISS1 (*p* = 0.169) between females and males affected by FASD.

The next step in data analysis was to evaluate the correlation between the clinical parameters of patients with FASD and the levels of the hormones studied (Table 5).

ASP was positively correlated with age (R = 0.434, *p* < 0.001, respectively), whereas α-MSH correlated negatively with age (R = −0.323, *p* = 0.011). We observed a weak negative correlation for α-MSH with BMI percentile (R = −0.288, *p* = 0.030). Furthermore, we found a weak negative correlation of NPY and α-MSH levels with HbA1c in the serum of patients with FASD (R = −0.356, *p* = 0.008, R = −0.302, *p* = 0.033, respectively). We also found a positive association between ACTH level and ASP (R = 0.380, *p* = 0.022). In addition, we analyzed the correlations between the hormones tested, which revealed a strong positive association between POMC [25] and ASP (R = 0.691, *p* < 0.001). ASP was also negatively correlated with the level of α-MSH (R = −0.316, *p* = 0.013). None of the hormones studied was correlated with NY and KISS1. There was also no correlation between the markers of the lipid profiles and the hormones analyzed.

## 4. Discussion

Our study describes, for the first time, serum levels of hormones regulating energy metabolism and nutrition, such as kisspeptin, neuropeptide Y, Agouti signaling protein, and alpha-melanocyte-stimulating hormone in patients with fetal alcohol spectrum disorders. Despite significant differences in BMI and growth retardation compared to healthy controls, there were no significant differences in the levels of the hormones studied in patients with FASD. This syndrome manifests itself in various ways, including poor growth, poor weight gain, improper eating patterns, altered appetite regulation, and nutritional deficits [14].

A key structure involved in maintaining energy balance is the arcuate nucleus of the hypothalamus, which contains two distinct populations of neurons: the neurons that suppress appetite (anorexigenic), proopiomelanocortin (POMC), and the neurons that increase appetite (orexigenic), neuropeptide Y /Agouti-related peptide (AgRP) neurons [26,27]. POMC and NPY form specific networks most often investigated in relation to energy intake. Both send messages on energy storage levels and nutritional status through leptin and insulin, mediated by specific receptors localized in POMC and NPY neurons, which are found primarily in the arcuate nucleus [28]. Furthermore, NPY in humans is synthesized in subcutaneous and visceral adipose tissues and is the most potent orexigenic peptide found in the brain that stimulates appetite, decreases thermogenesis, and increases plasma insulin and corticosterone levels [29]. NPY is also involved in the development of cardiovascular diseases, anxiety disorders, and posttraumatic stress disorders [30,31,32]. In addition, NPY may play a key role in modulating the development of alcohol dependence [33]. In our study, there were no differences in NPY levels between FASD and healthy participants, as well as between FASD subgroups and sex. Similar to our results, there were no statistically significant differences in neuropeptide Y levels between men and women in the group of healthy subjects [34]. There were also no differences in plasma NPY levels in bulimia patients compared to controls of the same age and weight, but elevated levels of NPY were also observed in bulimia [35,36]. Other studies revealed higher plasma levels of NPY in obese and overweight patients compared to controls, as well as in obese and nonobese women with polycystic ovary syndrome [37,38]. Recent studies in an animal model have shown that prenatal alcohol exposure leads to reduced feeding at all stages of development of *Drosophila melanogaster* due to reduced or eliminated neuropeptide signaling [39], but these findings were not confirmed in our study. Another study in rats revealed that hypothalamic NPY mRNA levels in the offspring of normally nourished dams resulted in lower levels of orexigenic factor compared to the offspring of undernourished dams [40]. NPY also affects lung growth after intrauterine growth restriction, which is often caused by maternal alcohol consumption during pregnancy [41,42].

α-MSH is a neuropeptide derived from POMC, which activates melanocortin receptors (MC4 and MC3) to regulate energy balance by stimulating energy expenditure and inhibiting appetite [26]. It is widely expressed in several tissues and organs and plays an important role in many biological processes, such as weight regulation, energy metabolism, sexual activity, and exocrine secretion [43]. This hormone α-MSH is also involved in the rewarding and addictive effects of ethanol [44]. However, the detailed action of peripheral α-MSH in energy homeostasis remains unclear. Chronic consumption of ethanol in rats significantly altered α-MSH levels, but the results are not consistent and showed different directions of changes [45,46]. We did not find any difference in the concentration of α-MSH between FASD patients and healthy participants. Plasma α-MSH level was elevated in obese men compared to nonobese individuals. Furthermore, α-MSH concentration was not affected by any changes in energy balance and weight loss [47,48]. Decreased serum α-MSH concentrations were found in patients with osteonecrosis of the femoral head and people with craniocerebral injury compared to healthy controls [49,50]. In our study, the concentrations of the α-MSH were independent of sex, in contrast to previous results in which levels of α-MSH were higher in males vs. females [51]. The severity of the disease may affect α-MSH levels, as the difference tends to be statistically significant depending on the type of FASD and was higher in FAS. Similar to our results, no differences in α-MSH levels have also been reported in groups of overweight and normal-weight individuals in both male and female populations [37].

Human ASP is highly expressed in adipose tissue, where it is a competitive antagonist of α-MSH for binding to the receptor [52]. AGRP and ASP are two of the most powerful appetite stimulators and express similar physiological functions. They might affect eating behavior, energy expenditure, adipocyte differentiation, and human pigmentation, but their role is not fully understood [53]. Overexpression of ASP has been proven to cause human obesity in childhood due to its orexigenic effects [54]. The study in cattle also confirmed associations between ASP expression and fat deposition [55]. Our previous study has shown that the concentration of ASP is reduced in serum from cystic fibrosis patients, whose nutritional status and BMI were lower compared to healthy controls [56]. However, we did not observe statistically significant differences in ASP levels between patients with FASD and the controls. The sex and type of FASD also had no effect on ASP levels.

Kisspeptin is another hypothalamic neuropeptide involved in the neuroendocrine regulation of gonadotropin-releasing hormone (GnRH) secretion [57]. It controls the onset of puberty, gonad development, trophoblast invasion, pregnancy, and lactation [58]. Reproduction is a task that requires adequate energy resources; therefore, control of reproduction and metabolism simultaneously is important for the survival of offspring and mother. In states of severely altered energy balance, fertility is altered, as well as the expression of kisspeptin in the arcuate nucleus of the hypothalamus [59]. Due to hypothalamic hypogonadism, it is frequently found in the case of disturbances in energy balance (positive and negative), such as anorexia nervosa or obesity [60].

The role of kisspeptin in the regulation of reproductive system function is fairly well understood, but its involvement in the regulation of energy balance is poorly characterized [61,62]. Recent studies have suggested that the dysregulation of KISS and other adipokines may be relevant in the pathogenesis of various diseases, such as obesity, diabetes, and sepsis [63,64]. Serum KISS concentrations were found to increase significantly in obese/overweight girls and obese men compared to the controls [65,66]. KISS1 probably does not act within the hypothalamus on its own but rather interacts with other neuropeptides, such as neurokinin B, dynorphin A, POMC, AgRP, and NPY [64,67,68]. KISS1 neurons are anatomically connected and can stimulate anorexigenic POMC neurons and indirectly inhibit orexigenic NPY neurons [69]. We found no differences in KISS levels in FASD participants compared to healthy volunteers, as well as between FASD and sex subgroups.

Several studies underlined the role of KISS1 in the development of central precocious puberty [70,71]. In individuals with FASD, puberty is often delayed and is considered one of the common clinical features of the disorder, but these symptoms are probably not directly associated with KISS1 levels [72]. However, studies on animal models have shown that prenatal alcohol exposure can disrupt the kisspeptin system and have adverse effects on the function of the hypothalamic−pituitary−gonadal axis that can contribute to delayed puberty in rats [73,74]. KISS1 shows a marked variability in expression and circulating levels between males and females [75]. We have not confirmed these findings in our study, probably because sex-specific differences in KISS1 levels are observed above the age of 12, much higher than the mean age of the study group [66,76]. Elevated plasma KISS1 levels were also found in boys and girls compared to adults, with a peak during puberty [77].

The correlations between hormone levels and clinical parameters of FASD patients were also evaluated. To our knowledge, no studies have investigated the correlation between NPY, ASP, α-MSH, KISS1, and clinical data in FASD. We found positive associations of ASP with age and ACTH level, but so far, no correlation between ASP and patient clinical data has been described in the literature, except our recent study in cystic fibrosis patients, which did not show a correlation with age or other clinical parameters [56]. We also found a strong positive correlation between ASP and POMC (R = 0.691, *p* < 0.001) and negative with α-MSH (R = −0.316, *p* = 0.013). ASP—orexigenic and α-MSH— anorexigenic factors compete for the availability of MC receptors, so the opposite direction of correlation is reasonable, but the positive association with POMC is less clear and might be explained by the involvement of POMC in more biological functions than regulation of energy homeostasis. We have found a negative correlation of α-MSH with age and BMI percentile as well as a positive correlation with HbA1c. It has been reported, that, contradictory to our results, the plasma level of α-MSH was positively correlated with BMI (R = 0.560, *p* < 0.05) and insulin levels (R = 0.528, *p* < 0.05) in obese men [47]. Another study has shown that serum α-MSH levels did not correlate with body composition parameters and fasting insulin levels [78]. Furthermore, the negative correlation between α-MSH and BMI has previously been demonstrated [79]. Similar to previous results. α-MSH did not correlate with NPY and any other hormones except ASP [53]. However, the positive correlation of plasma α-MSH level (R = 0.556, *p* < 0.01) with AgRP, which has a similar biological function as ASP, but our findings in relation to ASP were different (R = −0.316, *p* = 0.013) [80].

In our study, NPY was not correlated with the anthropological, functional, and biochemical parameters of the patients, nor with any hormones studied, except for a moderate negative association with HbA1c (R = −0.356, *p* = 0.008). Several studies have shown an inhibitory effect of cigarette smoking on brain NPY levels, leading to weight loss [81,82]. NPY is also involved in the prevention of negative consequences of stress, which is a factor that contributes to neurodegenerative diseases such as Alzheimer’s, Parkinson’s, and Huntington’s disease [83]. Previous studies revealed that NPY levels positively correlate with the age of the patients (R = 0.43, *p* = 0.001) [84]. Similar to our results, earlier studies did not find a correlation between NPY and BMI, nor between the waist/hip ratio [34]. Nevertheless, another study found that plasma NPY levels decreased significantly with age (R = −0.232, *p* = 0.038) and were not related to BMI [85].

KISS1 was not correlated with any of the parameters studied. Similar to our results, no correlation was found between kisspeptin and BMI in serum levels of girls with central precocious puberty [86]. No correlation has also been reported between KISS1 and HOMA-IR and insulin in obese and overweight children [87]. However, contradictory results (positive correlation of KISS1 with body mass, BMI, plasma insulin, and HOMA-IR) in 40 male subjects were also observed [65]. Finally, we have not found any correlation between the hormones studied and markers of the lipid profile, which may indicate that these factors are not predictors of serum levels of these hormones in patients with FASD.

Even though our study describes for the first time the effect of prenatal alcohol exposure on hormonal regulation of feeding and nutrition, its results must be considered preliminary and subject to some uncertainty. One of the study’s limitations is that the number of participants was insufficient to definitively conclude that prenatal alcohol exposure does not alter the secretion of ASP, α-MSH, NPY, and KISS1. Second, the age range of the participants was quite broad, which may have affected the final conclusions. Third, the number of participants in the control group was markedly lower than the FASD group, and the number of males was more than double that of females, which could also disrupt the reliability of the statistical analysis. Finally, there are a very small number of manuscripts describing hormonal regulation in FASD. Therefore, when writing the discussion, we referred to studies on other species or disorders with different etiologies than FASD, which described the role of the hormones studied in the regulation of nutrition and the effect of alcohol on their secretion.

## 5. Conclusions

In summary, we investigated 57 patients with FASD and 23 healthy controls. All hormones studied were analyzed for the first time in individuals with confirmed prenatal alcohol exposure. A comparison of the levels of ASP, α-MSH, NPY, and KISS1 was made, also in the FAS vs. ND PAE subgroups, as well as in males and females. We have found no differences in the levels of the hormones tested between affected individuals and the controls and between subgroups of FASD, such as FAS and ND PAE. In addition, sex did not affect hormone levels. However, we identified some correlations between hormone concentrations and parameters that describe the clinical status of patients with FASD. Most of them were related to ASP, which was positively correlated with age and ACTH. We have also found a negative correlation of α-MSH with age, BMI percentile, and HbA1c. Furthermore, we found a weak negative correlation of NPY with HbA1c. Although FASD is associated with impaired proper child growth and development, including nutrition and puberty onset, we did not identify differences in the levels of the hormones studied, which may suggest that prenatal alcohol exposure does not affect the levels of these metabolites.

## Figures and Tables

**Table 1:** Baseline demographic and clinical data of the study participants.

		FASD	Healthy Controls	*p* Value
Sex (F/M)		29/28	7/16	
Age (years)	Mean ± SD	8.01 ± 3.95	7.45 ± 5.12	0.526
	range	1.83–16.5	0.42–17	
BMI percentile	Mean ± SD	32.29 ± 31.79	60.71 ± 27.03	**0.037**
	range	0.1–99.9	12–99	
Clinical laboratory markers				
Cholesterol (mg/dL) Norm < 190	Median	158	155	0.929
	Range	76–244	126–191	
LDL (mg/dL) Norm < 135	Median	90.5	95	0.848
	Range	31–163	72–104	
HDL (mg/dL) Norm > 40	Median	53	53	0.948
	Range	24–108	42–59	
Triglycerides (mg/dL) Norm < 150	Median	67	65	0.828
	Range	30–241	38–141	
Glucose (mg/dL) Norm (70–99)	Median	85.5	87	0.829
	Range	72–99	6–94	
Insulin (mIU/mL) Norm < 15	Median	5.03	2.05	0.156
	Range	1.41–16.46	1.0–9.03	
HbA1c (%) Normal range (4–6)	Median	5.38	5.41	0.801
	Range	4.81–5.86	5.26–5.55	
HOMA-IR Norm < 2.5	Median	1.05	0.78	0.223
	Range	0.27–3.62	0.21–2.03	

BMI percentile—body mass index percentile, LDL—low-density lipoprotein, HDL—high-density lipoprotein, HbA1c—glycated hemoglobin, HOMA-IR—homeostasis model assessment of insulin resistance; data are presented as median and range or mean, and standard deviation (SD); differences between means were analyzed using the Mann−Whitney U test. Statistically significant differences were bold.

**Table 2:** Baseline demographic and clinical data of FASD subgroups.

		FAS	ND-PAE	*p* Value
Sex (F/M)		14/12	15/16	
Age (years)	Mean ± SD	7.91 ± 4.77	8.13 ± 3.32	0.843
	range	0.42–16	2.08–13.5	
BMI percentile	Mean ± SD	22.12 ± 27.51	42.04 ± 33.02	**0.02**
	range	0.1–78	0.1–99.9	
Clinical Laboratory Markers				
Cholesterol (mg/dL) Norm < 190	Median	154.	161	0.110
	Range	76–238	114–244	
LDL (mg/dL) Norm < 135	Median	86	75	0.365
	Range	31–143	114–244	
HDL (mg/dL) Norm > 40	Median	49.5	53	0.382
	Range	33–80	24–108	
Triglycerides (mg/dL) Norm < 150	Median	64	75	0.607
	Range	30–229	34–241	
Glucose (mg/dL) Norm (70–99)	Median	82	87	0.211
	Range	72–99	74–99	
Insulin (mIU/mL) Norm < 15 mIU/ml	Median	5.1	4.22	0.623
	Range	1.41–16.46	1.56–13.97	
HbA1c (%) Normal range (4–6)	Median	5.24	5.45	**0.039**
	Range	4.81–5.86	4.89–5.85	
HOMA-IR Norm < 2.5	Median	1.07	1.29	0.79
	Range	0.27–3.62	0.31–3.53	

BMI percentile—body mass index percentile, LDL—low-density lipoprotein, HDL—high-density lipoprotein, HbA1c—glycated hemoglobin, HOMA-IR—homeostasis model assessment of insulin resistance; data are presented as median and range or mean, and standard deviation (SD); comparison between means were analyzed using the Mann−Whitney U test. Statistically significant differences were bold.

**Table 3:** Hormone levels in subgroups of patients with FASD.

		FAS	ND-PAE	*p* Value
Agouti-signaling protein (pg/mL)	Median	14.02	14.59	0.59
	Range	12.22–35.18	12.18–32.28	
Neuropeptide Y (pg/mL)	Median	316.22	303.82	0.278
	Range	216.75–429.7	217.4–430.3	
α-MSH (pg/ml)	Median	15.44	13.65	0.071
	Range	12.16–18.23	10.59–17.99	
KISS1 (ng/mL)	Median	2.03	2.16	0.585
	Range	1.18–4.04	1.09–3.65	

Data are presented as median and range; comparisons between means were analyzed using the Mann−Whitney U test.

**Table 4:** Hormone levels by sex of patients with FASD.

Hormone	FASD			
		Female	Male	*p* Value
Agouti-signaling protein (pg/mL)	Median	13.86	14.27	0.856
	Range	12.22–32.28	12.18–35.18	
Neuropeptide Y (pg/mL)	Median	306.9	307.25	0.596
	Range	217.4–429.7	216.75–450.4	
α-MSH (pg/mL)	Median	15.43	14.27	0.173
	Range	11.85–18.23	10.59–17.99	
KISS1 (ng/mL)	Median	7.3	9.8	0.169
	Range	3.5–18.9	5–26	

Data are presented as median and range; comparisons between means were analyzed using the Mann−Whitney U test.

**Table 5:** Spearman rank correlation coefficients (R) and *p*-values between hormone concentrations and clinical features of the patients studied.

		Age	BMI per	Cortisol	ACTH	Ch	LDL	HDL	TGL	Glu	Ins	HOMA-IR	HbA1c	KISS1	ASP	NPY	α-MSH	POMC
KISS1	R	−0.119	0.244	−0.008	0.095	0.115	0.066	−0.025	0.151	−0.151	0.04	0.049	0.064		0.046	0.042	−0.065	0.121
	*p*	0.359	0.065	0.952	0.583	0.376	0.613	0.849	0.246	0.257	0.772	0.723	0.644		0.722	0.748	0.618	0.348
Agouti- signaling protein	R	0.434	0.228	0.015	0.380	0.211	0.163	0.094	0.097	0.149	0.233	0.250	0.218	0.046		−0.164	−0.316	0.691
	*p*	**<0.001**	0.086	0.913	**0.022**	0.103	0.209	0.47	0.459	0.264	0.089	0.068	0.113	0.722		0.203	**0.013**	**<0.001**
Neuropeptide Y	R	−0.109	−0.163	0.036	0.092	−0.011	−0.045	−0.078	0.006	−0.23	−0.137	−0.152	−0.356	0.042	−0.164		0.025	−0.145
	*p*	0.397	0.220	0.787	0.593	0.931	0.733	0.548	0.966	0.082	0.323	0.273	**0.008**	0.748	0.203		0.846	0.26
α-MSH	R	−0.323	−0.288	0.096	−0.134	−0.202	−0.111	−0.17	−0.035	−0.213	−0.12	−0.150	−0.302	−0.065	−0.316	0.025		−0.171
	*p*	**0.011**	**0.030**	0.475	0.442	0.123	0.4	0.195	0.802	0.111	0.392	0.284	**0.033**	0.618	**0.013**	0.846		0.187

BMI per—body mass index percentile, ACTH—adrenocorticotropic hormone, Ch—cholesterol, LDL—low-density lipoprotein, HDL—high-density lipoprotein, TGL—triglycerides, Glu—glucose, Ins—insulin, HOMA-IR—homeostasis model assessment of insulin resistance, HbA1c—glycated hemoglobin. Statistically significant differences were bold.

## Data Availability

The data presented in this study are available on request from the corresponding author.
